# Feasibility and repeatability of ultrasound-guided surface electroenterography to measure colonic slow wave motility in healthy adults

**DOI:** 10.1186/s12876-024-03196-w

**Published:** 2024-03-18

**Authors:** Nick H. Rolleman, Iris M. Visser, Willemijn M. Klein, Michel J.A.M. Van Putten, Ivo De Blaauw, Sanne M.B.I. Botden

**Affiliations:** 1 Department of Pediatric Surgery, Radboud University Medical Centre– Amalia Children’s Hospital, Geert Grooteplein Zuid 10, 6500 HB Nijmegen, Postal box 9101, The Netherlands; 2 Technical Medicine, University of Twente, Enschede, The Netherlands; 3 Department of Medical Imaging, Radboud University Medical Centre, Nijmegen, The Netherlands; 4 Department of Clinical Neurophysiology, University of Twente, Enschede, The Netherlands

**Keywords:** Electroenterography, Non-invasive, Colon, Motility, Gastrocolic reflex

## Abstract

Surface electroenterography is a potential non-invasive alternative to current diagnostics of colonic motility disorders. However, electrode positioning in electroenterography is often based on general anatomy and may lack generalizability. Furthermore, the repeatability of electroenterography measurements is unknown. This study aimed to evaluate ultrasound-guided electrode positioning for electroenterography measurements and to determine the repeatability of those measurements. In ten healthy adults, two electroenterography procedures were performed, consisting of fasting, ultrasound-guided electrode localization and two 20-minute electroenterography recordings separated by a meal. The dominant frequency, the mean power density (magnitude of colonic motility) and the power percent difference (relative pre- to postprandial increase in magnitude) were determined. Repeatability was determined by Lin’s concordance correlation coefficient. The results demonstrated that the dominant frequency did not differ between pre- and postprandial recordings and was 3 cpm, characteristic of colonic motility. The mean power density increased between the pre- and postprandial measurements, with an average difference of over 200%. The repeatability of both the dominant frequency and power density was poor to moderate, whereas the correlation coefficient of the power percent difference was poor. Concluding, ultrasound-guided surface electroenterography seems able to measure the gastrocolic reflex, but the dissatisfactory repeatability necessitates optimization of the measurement protocol.

## Background

A recent multi-national, survey-based study showed that among 73.076 adults, more than 30% met the criteria for having at least one functional bowel disorder, e.g. irritable bowel syndrome, functional constipation or slow transit constipation [1, 2]. Such disorders, in which intestinal motility is abnormal, are often accompanied by difficulty with defecation, abdominal pain and may drastically decrease the quality of life [2]. 

Assessment of intestinal motility often involves multiple diagnostic tools, including clinical history, physical examination, blood tests, transit studies, barium enemas, colonoscopies and colonic manometries [2, 3]. Many of these procedures are invasive, require complex skill or are pain- or shameful and cannot always provide a clear-cut diagnosis [2, 4–6]. This advocates for a more adequate and non-invasive method for the diagnosis of intestinal motility disorders.

One such method, overcoming some of the mentioned challenges, may be surface electroenterography (sEEnG), which involves placing electrodes on the abdomen to measure colonic slow wave activity, oscillating at 2–6 cycles per minute (cpm) [7, 8]. SEEnG is unique in the sense that it is both non-invasive and aims to directly reflect the functional aspect of the colon, whereas other diagnostic tools are either invasive or indirectly assess colonic activity. Previously, researchers have performed measurements using sEEnG and demonstrated that the gastrocolic reflex, which is the increase in colonic activity upon food entering the stomach, could be measured using sEEnG [9–12]. However, the position of the electrodes was based on general anatomy, thereby impeding the inter-subject comparability and thus the generalizability of the results. Furthermore, not much is currently known about the repeatability of sEEnG measurements. This study aimed to evaluate the use of ultrasound-guided electrode positioning for sEEnG measurements and to determine the repeatability of these measurements in healthy adults.

## Methods

### Study design and setting

In this observational, cross-sectional study, pre- and postprandial sEEnG measurements were performed twice, two weeks apart, in adults without gastrointestinal complaints. The study was conducted in the Netherlands and the inclusion period was from February 2021 to March 2022. Approval from the local medical ethical committee was obtained (NL75302.091.20).

### Participants

Adult participants (> 18 years old) were included in this study. Exclusion criteria were a body mass index (BMI) of > 27 kg/m^2^, pregnancy, diabetes, any food intolerances, presence of an intestinal stoma, use of continuous tube feeding, the presence of gastrointestinal diseases or complaints and the use of laxatives in the past two years. Participants were recruited from medical students, the Dutch patient organization for Hirschsprung’s disease and parents of hospitalized children. All participants signed an informed consent form prior to participation.

### Protocol

**Fig. 1:**
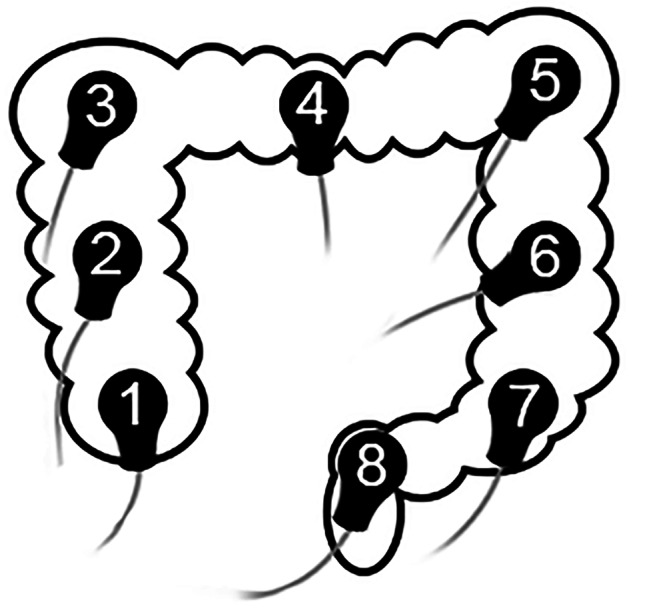
Electrode positions, targeting different segments of the colon. 1 = cecum, 2 = mid-ascending, 3 = hepatic flexture, 4 = mid-transverse, 5 = splenic flexture, 6 = mid-descending, 7 = sigmoid, 8 = rectum

The measurement protocol comprised two sEEnG procedures. Before the first sEEnG procedure, participants fasted for at least four hours. Subsequently, eight surface electrodes were placed on the abdomen by a radiologist, using ultrasound-guidance, with the participant in supine position. The radiologist was asked to locate the colonic segments as shown in Fig. 1. A ground electrode was placed on the middle of the right clavicle. The electrode positions were documented using measuring tape and anatomical landmarks, e.g. the anterior superior iliac spines and the umbilicus, which allowed identical electrode placement during the second sEEnG procedure. Next, a baseline sEEnG recording of 20 min was obtained, after which the participants consumed a meal of their own choice consisting of at least 1/6th of their daily recommended calorie intake based on the advice of The Netherlands Nutrition Centre [13]. Directly after the meal, another 20-minute sEEnG recording was started to capture the colonic activity initiated by the gastrocolic reflex.

The second sEEnG procedure was performed approximately two weeks later, at the same time of the day to avoid the possible influence of the circadian rhythm [14]. This procedure was identical to the first, except that the electrodes were positioned using the descriptions of the locations obtained during the first procedure instead of by ultrasound. It was ensured that the duration of fasting as well as the meal composition and duration were comparable in both procedures. All measurements were acquired using silver-silver chloride electrodes (30 × 24 mm, Covidien, Massachusetts, USA), an amplifier (Porti7, TMSi, Oldenzaal, the Netherlands) and the Polybench software (version 1.34.0, TMSi, Oldenzaal, the Netherlands). The signals were sampled at 2048 Hz. Additionally, participant characteristics (age, sex, length, weight and BMI) and defecation habits (defecation frequency, average Bristol stool scale and time since last defecation) were obtained [15]. Defecation frequency and average Bristol stool scale were obtained during the sEEnG procedures by asking the subjects to estimate their average defecation frequency and Bristol stool scale.

### Signal analysis

Signal preprocessing was performed separately but identical for the pre- and postprandial data and consisted of resampling at 20.48 Hz and applying a fourth order Butterworth bandpass filter with cut-off frequencies of 1.5 and 10 cpm. Next, the artifact rejection methodology proposed by Gharibans et al. was applied [16]. This methodology was initially developed for gastric slow wave recordings, but because the gastric slow wave frequency is comparable to the colonic slow wave frequency, it is justified to use it for sEEnG recordings. Finally, power spectra of the signals were obtained using Welch’s method with a 240-second Hann-window and 50% overlap.

**Fig. 2:**
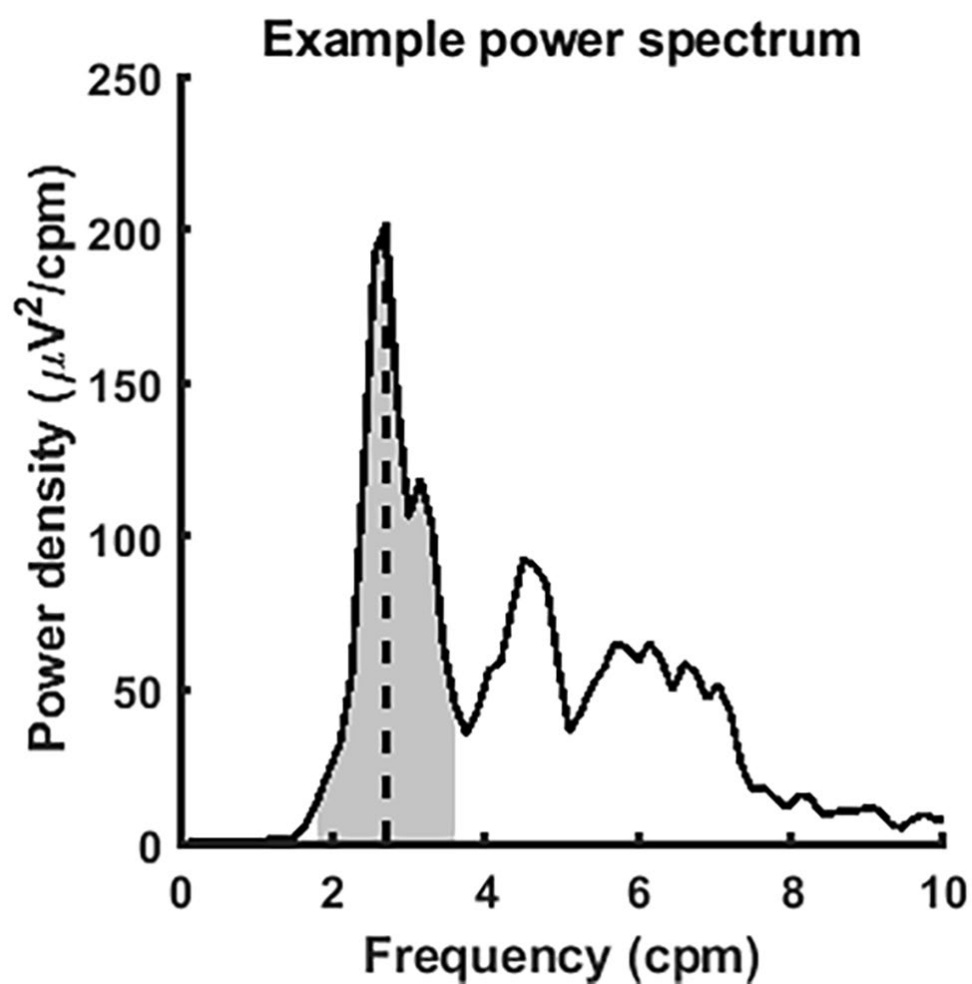
Example of a representative power spectrum (black curve). The dominant frequency is the frequency at maximal power density (dotted line) and the MPD is the average power density around the dominant frequency (average height of the grey area)

To quantitatively describe the sEEnG recordings, three features were extracted from the power spectra, see Fig. 2. The first feature was the dominant frequency. This was defined as the frequency with the highest power spectral density. The second feature, the mean power density (MPD) expressed in µV^2^/cpm, was calculated as the average power spectral density in a 2 cpm bandwidth around the dominant frequency and represented the magnitude of colonic activity. Finally, the activity increase between the pre- and postprandial measurements was expressed as a percentage and denoted as the percent power difference (PPD). Each feature was averaged over the proximal two, the middle three and distal three electrodes per participant to gain insight in the regional variations of the sEEnG measurements. All signal analyses were performed using MATLAB (Version R2018a, The Mathworks Inc., Natick, Massachussets).

### Statistical analysis

All values are presented as mean ± standard deviation or median (first quartile– third quartile) and all sEEnG features are analyzed per colonic segment. Differences in the procedure related characteristics between the first and second sEEnG procedure were tested using paired t-tests. Differences in sEEnG features between pre- and postprandial measurements were tested using Wilcoxon matched-pair signed-rank tests. Regional variations between the sEEnG features were assessed using Friedman’s test. To test for interaction between the meal states and the colonic segments on the dominant frequency and the MPD, a 2-way ANOVA test was used. The repeatability of the sEEnG measurements was determined using Lin’s concordance correlation coefficient (LCCC) and its 95% confidence interval (CI) [17]. The LCCC is a measure of agreement between two recordings and ranges from − 1 to 1, with a perfect correlation at 1, no correlation at 0 and a perfect inverse correlation at -1. The LCCC values will be marked as very high (> 0.9), high (0.7–0.9), moderate (0.5–0.7), low (0.3–0.5) or poor (< 0.3) [18]. For all tests, *p*-values < 0.05 were considered statistically significant. The statistical analyses were performed using IBM SPSS Statistics for Windows (Version 25.0, IBM Corp., Armonk, New York).

## Results

In total, ten participants were included, of whom five were women. Their mean age was 27 ± 9 years (range 19–47) and their mean BMI was 23.2 ± 2.7 kg/m^2^ (range: 18.8–26.5). The participant’s average Bristol stool scale was 3 ± 1. Their defecation frequency was 7 ± 2 times per week. The time between the first and the second sEEnG procedure was 13 ± 5 days and, averaged over both procedures, the time since last defecation before the procedure was 10.0 ± 7.4 h and the duration of fasting was 5.4 ± 1.1 h. During the procedures, the participants consumed a meal containing 520 ± 171 kcal and spent 13.9 ± 4.9 min eating it. These values did not differ between the first and second procedure and all participants completed both procedures.

**Fig. 3:**
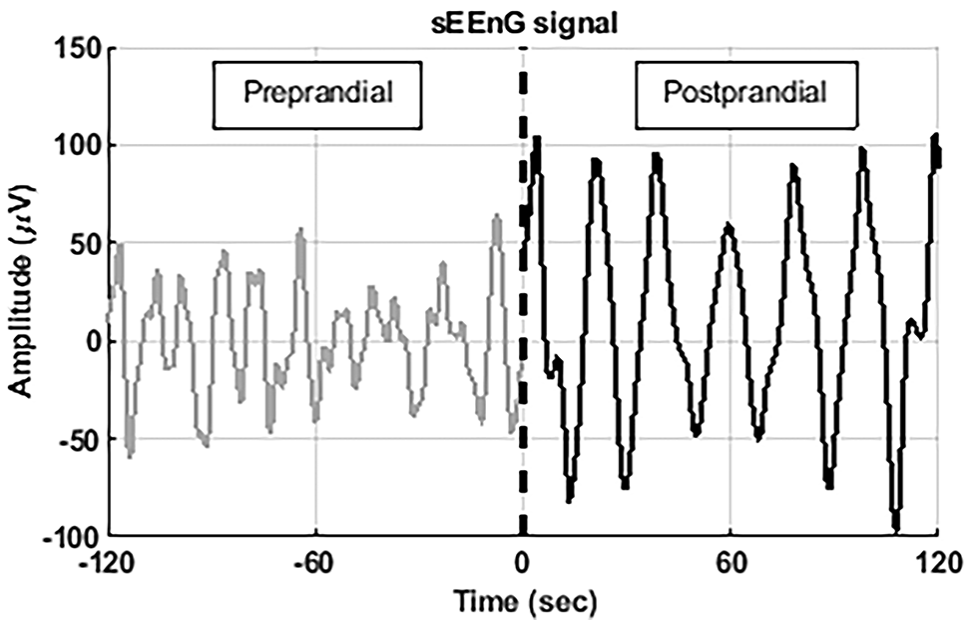
Representative sEEnG signal segment. The dotted line indicates the meal. The postprandial signal (black) clearly exhibits the ∼ 3 cpm colonic slow waves and has a higher amplitude than the preprandial signal (grey)

**Fig. 4:**
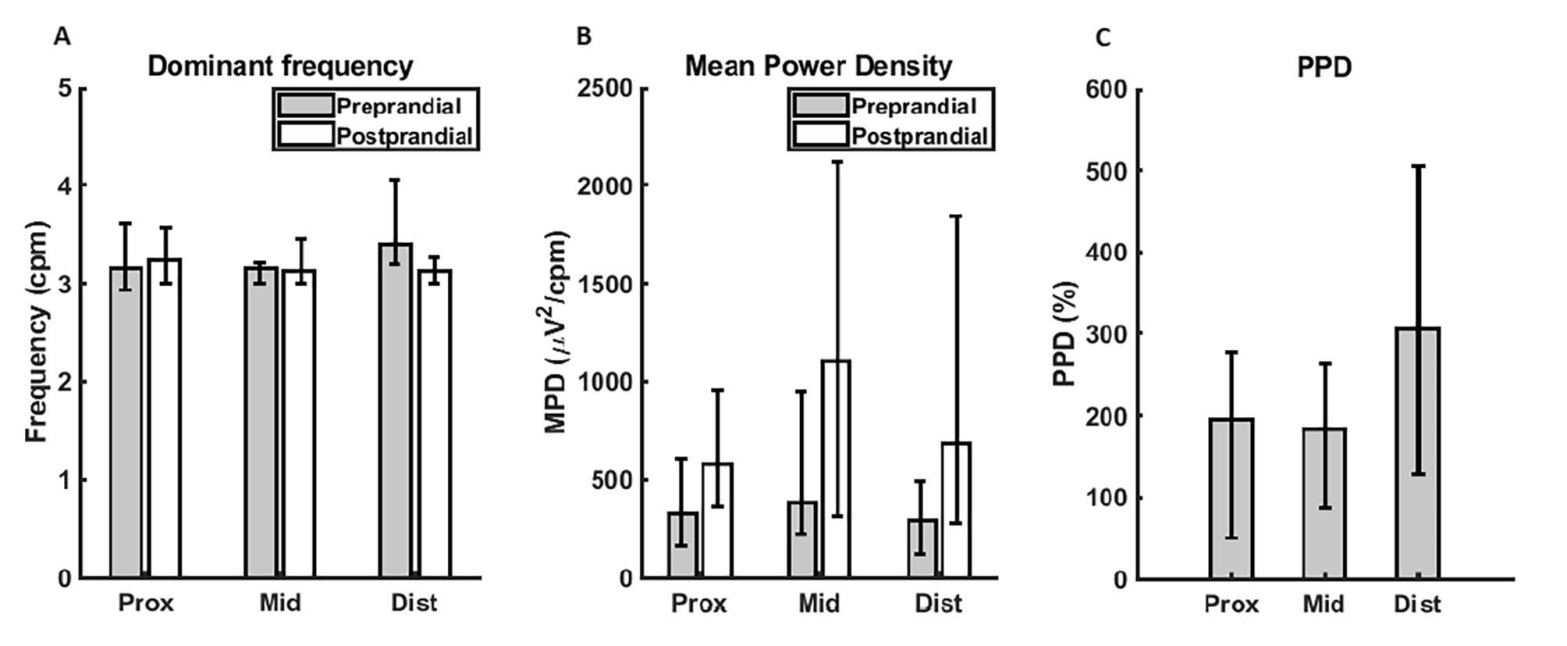
Preprandial (grey) and postprandial (white) values of the dominant frequency (**A**), mean power density (**B**) and power percent difference (**C**) per colonic segment of the first sEEnG recording

To illustrate, Fig. 3 shows a representative sEEnG signal segment of one electrode. In Fig. 4, the pre- versus postprandial dominant frequency, MPD and PPD for the first sEEnG procedure are shown per colonic segment and averaged over all subjects. In all segments, the dominant frequency was around 3 cpm for both the pre- and postprandial recordings. However, the dominant frequency differed between pre- and postprandial recordings in the distal colon, with the preprandial dominant frequency being higher than the postprandial dominant frequency, 3.4 cpm (3.2–4.1) vs. 3.1 cpm (3.0–3.3), *p* = 0.017. Nevertheless, when taking the possible interaction between meal state and colonic segment in consideration, no significant differences in dominant frequency were found (*p* = 0.287). Regarding MPD, the postprandial MPD was significantly higher than the preprandial MPD in all colonic segments, with *p*-values of 0.017, 0.005 and 0.007 for the proximal, middle and distal segment respectively. Comparing the dominant frequency and MPD between the colonic segments did not reveal significant differences. This was also reflected in the 2-way ANOVA test, showing that the MPD was significantly different per meal state (*p* = 0.010), but not per colonic segment (*p* = 0.180). Nevertheless, note that the median postprandial MPD is nearly twice as high as in the middle colon compared to the proximal and distal colon. Furthermore, the PPD in the distal colon is more than 1.5 times as high as the PPD in the proximal and middle colon.

**Fig. 5:**
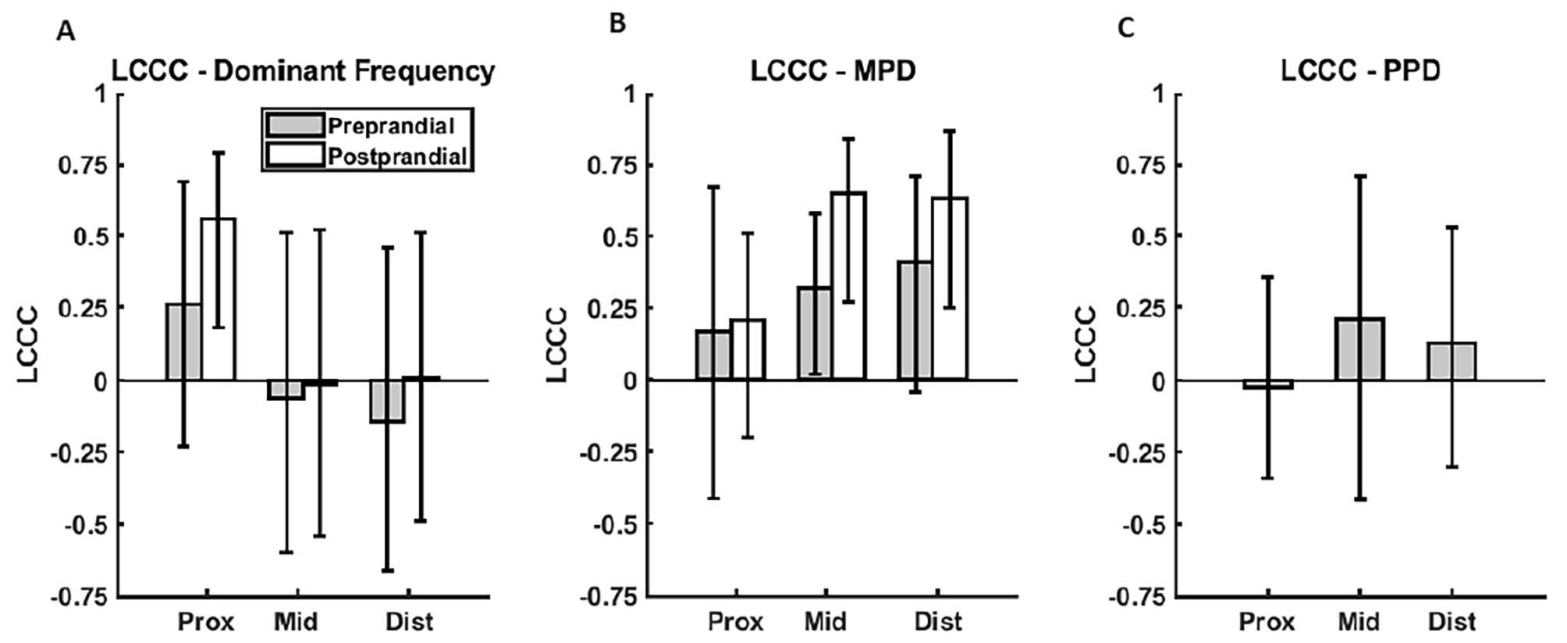
Preprandial (grey) and postprandial (white) Lin’s concordance correlation coefficients for the dominant frequency (**A**), mean power density (**B**) and power percent difference (**C**) per colonic segment

Table 1 shows the absolute values of all sEEnG features per colonic segment for both sEEnG procedures and Fig. 5 shows the corresponding repeatability. The dominant frequency, Fig. 5A, showed poor repeatability for both pre- and postprandial recordings along the entire colon, except for the postprandial recordings in the proximal colon, which showed moderate repeatability (0.56 (0.18 − 0.79)). Averaged over all segments and both pre- and postprandial recordings, the absolute difference in dominant frequency between the first and second recording is 0.5 cpm. For MPD, see Fig. 5b, repeatability was poor for both pre- and postprandial recordings in the proximal colon. For the middle and distal colon, MPD repeatability was low for the preprandial recordings and moderate for the postprandial recordings. Note that the repeatability tended to be higher for the postprandial MPD?s when compared to the preprandial MPD?s. The repeatability of the PPD, shown in Fig. 5C, was poor for all colonic segments.

**Table 1:** Absolute values of pre- and postprandial sEEnG features for the first and second procedure

			First sEEnG procedure	Second sEEnG procedure
Preprandial	DF	Proximal	3.2 (2.9–3.6)	3.5 (3.0–4.1)
	(cpm)	Middle	3.2 (3.0–3.2)	3.1 (2.9–3.4)
		Distal	3.2 (3.4–4.1)	3.2 (2.9–3.5)
	MPD	Proximal	329 (158–605)	286 (195–688)
	(µV^2^/cpm)	Middle	380 (225–951)	363 (264–758)
		Distal	293 (119–490)	274 (171–663)
Postprandial	DF	Proximal	3.2 (3.0–3.6)	3.3 (2.8–3.5)
	CPM	Middle	3.2 (3.0–3.5)	3.3 (3.1–3.3)
		Distal	3.1 (3.0–3.3)	3.3 _3.1–3.5)
	MPD	Proximal	582 (358–957)	700 (405–898)
	(µV^2^/cpm)	Middle	1105 (310–2122)	1015 (431–3200)
		Distal	680 (276–1845)	717 (382–1843)
	PPD	Proximal	195 (50–278)	68 (28–171)
	(%)	Middle	184 (86–262)	143 (84–206)
		Distal	306 (127–506)	181 (113–230)

## Discussion

In this study, ultrasound-guided sEEnG measurements of the gastrocolic reflex and its repeatability were researched. The results demonstrated that ultrasound-guided sEEnG measurements can adequately record colonic slow waves in healthy adults, because a clear increase in the postprandial activity with respect to the preprandial activity within the 2–6 cpm bandwidth was observed in all colonic segments [11, 19]. However, the repeatability of the sEEnG measurements was low to poor for most features in all colonic segments and was moderate at its best. Our findings suggest that it is feasible to measure colonic motility using surface electrodes placed under ultrasound guidance, but that the low repeatability, especially of the MPD and PPD as indicators of the gastrocolic reflex, limits the usability of the studied measurement protocol for adequate assessment of colonic motility.

Although pre- and postprandial sEEnG measurements have been performed before, the use of ultrasound to position the sEEnG electrodes has not been described previously. Homma et al. based their electrode positions on anatomical landmarks, whereas Erickson et al. completely covered the abdomen between the umbilicus and the upper region of the symphysis pubis with 30–32 electrodes [10, 11]. Similar to our study, both authors described an increase in postprandial activity with respect to the preprandial activity in the 2–6 cpm frequency bandwidth. However, they reported relative power increases of approximately 63% (48–114) and 36 ± 18%, which are substantially lower than the PPD values reported in this study, which were between 184 − 306% [10, 11]. The use of ultrasound-guided electrode localization may be largely responsible for these differences, because this ensures the electrodes are placed as close to the colon as possible. Nevertheless, the impact of variations in the measurement protocols, especially regarding meal content and measurement duration, cannot be fully dismissed.

Furthermore, repeatability of sEEnG measurements has not been determined appropriately before. In 2020, Axelrod et al. demonstrated that the dominant frequency and signal intensity of sEEnG were constant over a period of three years, but they only demonstrated this graphically [20]. The results of the current study provide new insights in the use of ultrasound to guide sEEnG electrode positioning as well as the repeatability of the sEEnG measurements.

The main strength of this study is that the sEEnG is recorded both pre- and postprandially. This allows for comparisons of the sEEnG features within each participant and eliminates the need to compare absolute values between individuals, which can vary substantially. Another strong point is that the circumstances for both sEEnG procedures, such as time of the day, hours of fasting and meal content and duration, were kept as identical as possible, which allowed adequate assessment of the repeatability of the sEEnG measurements. Additionally, the 20-minute period of pre- and postprandial recording should be sufficient to capture the gastrocolic reflex and show clear differences in colonic activity. Previous research showed that the gastrocolic reflex peaks approximately 20 min after the start of a meal and tapers off at 30 min after the start of the meal [19]. Taking into account that the subjects needed at least ten minutes to finish their meal, a 20 min postprandial recording was regarded sufficient. This is also confirmed by the presented data, showing a clear postprandial increase in colonic slow wave activity in 20-minute measurements. Nevertheless, as colonic motility is complex and, among others, affected by psychological, hormonal and immunological factors, including the anticipation of a meal, a 20-minute sEEnG recording may not adequately reflect the targeted state (i.e. fasting) [8, 21]. 

An important limitation of this study is that the electrical activity of the stomach was not taken into account. The slow wave frequency of the stomach is, similarly to colonic slow waves, around 3 cpm and also shows a postprandial power increase [22]. However, it is not plausible that all measured activity originated in the stomach, because the largest relative postprandial motility increase was observed in the distal colon and not in the middle, which is consistent with earlier research reporting slow wave activity to be most present in the distal colon [23]. Nevertheless, partial influence of gastric activity cannot be excluded based on the results. Another limitation may be that all subjects consumed different meals between the pre- and postprandial recordings, because it has been suggested in earlier studies that the number of calories and meal content may affect the gastrocolic reflex [24, 25]. However, this limitation does not apply to the repeatability measurements, because each subject consumed the same meal during both procedures. Furthermore, no ultrasound-guided electrode placement was applied for the second sEEnG recording. This may have affected the reproducibility of the recordings, because the colon is not entirely fixed within the abdomen. Nonetheless, if ultrasound-guided electrode placement would have been employed for the second sEEnG recording as well, the distance between electrodes and colon as well as the electrode position in relation to the stomach would still have varied between both recordings and thus affected reproducibility. A final limitation of the study may be that summary metrics were used to describe the dynamic process of colonic motility. Even though these summary metrics have proven to show the ability of sEEnG to measure the colonic reflex, it is still an oversimplification of the complex colonic physiology.

Despite these limitations, the results clearly demonstrated that ultrasound-guided sEEnG can measure colonic activity elicited by the gastrocolic reflex. This is not only based on the postprandial power increase in all colonic segments, but also on the fact that the PPD tended to be higher in the distal colon when compared to the proximal and middle colon, corresponding to findings in earlier studies regarding the gastrocolic reflex [19, 26]. The clinical importance of this finding, is that sEEnG may be of value in diagnosing colonic motility disorders. Research has shown that the interstitial cells of Cajal, an important factor affecting colonic slow wave activity, are involved in colonic motility disorders, meaning their function can possibly be assessed using sEEnG [27, 28]. 

Regarding repeatability, it was surprising that the pre- and postprandial LCCC’s of the dominant frequency were poor for most colonic segments, because the absolute differences between the first and second procedure were small. However, the LCCC is directly dependent on the interparticipant variability of the feature of interest, which was low for the dominant frequency, resulting in a poorer repeatability. Nevertheless, for all recordings, the dominant frequencies were around 3 cpm, characteristic for colonic motility, thus the poor repeatability of the dominant frequency is not assumed to be of any clinical relevance.

The poor to moderate repeatability of the MPD, however, may limit the usefulness of sEEnG measurements to assess colonic motility. Although the circumstances of the first and second sEEnG recording were kept as comparable as possible, aspects such as colonic contents, physiological state, hormonal levels and spontaneously occurring activity may have a significant impact on the measured colonic activity [8, 29]. Additionally, the dynamic nature of the gastrocolic response was not reflected in the MPD, which may have affected the repeatability as well. The repeatability of the MPD showed an interesting pattern, because the preprandial repeatability tended to be lower than the postprandial repeatability in each colonic segment. A viable reason for this observation is that the postprandial activity is mainly regulated by the consumption of the meal, which was comparable for both recordings, whereas preprandial activity does not primarily depend on one stimulus. Aside from the factors mentioned previously, the cephalic response may greatly influence colonic motility during the preprandial phase, which makes this phase more susceptible to variations than the postprandial phase [8, 21, 29]. Concerning PPD, its poor repeatability is not surprising, as this is influenced by the variability in both the pre- and postprandial MPD. Unfortunately, this may severely impede the use of our measurement protocol consisting of 20-minute pre- versus postprandial sEEnG measurements in the assessment of colonic motility.

Based on our findings, the current measurement protocol is unable to consistently measure colonic motility in response to a meal. Therefore, improvements in the protocol must be made prior to starting sEEnG studies in patients with colonic motility disorders. Important aspects in optimizing the measurement protocol are the timing and duration of the measurements, the contents of the meal, analysis of the dynamic properties of colonic motility and possibly the effect of the participant’s characteristics, e.g. sex and bodyweight (or BMI). It might also be rewarding to focus on other types of colonic motility which are not directly related to the gastrocolic reflex, such as recently described by Pervez et al. [23]. They studied high-resolution colonic manometries in healthy adults and found a cyclic motor pattern with a frequency of 11–13 cpm which was present throughout the entire colon, which presumably can also be recorded using sEEnG. To this end, longer sEEnG measurements are probably necessary, because this activity occurs either isolated or following a high amplitude pressure wave and are not related to the gastrocolic reflex [23]. 

## Conclusion

This study demonstrated that ultrasound-guided sEEnG is feasible and that sEEnG is capable of recording the increase in magnitude of colonic slow waves upon the gastrocolic reflex in healthy adults The poor to moderate repeatability of the different sEEnG features are limiting of the usability of the current measurement protocol to adequately access colonic motility. Therefore, further study to optimize the sEEnG measurement protocol and standardize the outcomes of the measurements is necessary.

## Data Availability

Data are available from the author upon reasonable request by sending an email to nickrolleman@outlook.com.

## Abbreviations

sEEnG: Surface electroenterography
cpm: Cycles per minute
BMI: Body mass index
MPD: Mean power density
PPD: Percent power difference
LCCC: Lin’s concordance correlation coefficient
